# Urupocidin C: a new marine guanidine alkaloid which selectively kills prostate cancer cells via mitochondria targeting

**DOI:** 10.1038/s41598-020-66428-5

**Published:** 2020-06-17

**Authors:** Sergey A. Dyshlovoy, Ekaterina K. Kudryashova, Moritz Kaune, Tatyana N. Makarieva, Larisa K. Shubina, Tobias Busenbender, Vladimir A. Denisenko, Roman S. Popov, Jessica Hauschild, Sergey N. Fedorov, Carsten Bokemeyer, Markus Graefen, Valentin A. Stonik, Gunhild von Amsberg

**Affiliations:** 1 Department of Oncology, Hematology and Bone Marrow Transplantation with Section Pneumology, Hubertus Wald-Tumorzentrum, University Medical Center Hamburg-Eppendorf, Hamburg, Germany; 2 G.B. Elyakov Pacific Institute of Bioorganic Chemistry, Far-East Branch, Russian Academy of Sciences, Vladivostok, Russian Federation; 3 School of Natural Sciences, Far Eastern Federal University, Vladivostok, Russian Federation; 4 Martini-Klinik, Prostate Cancer Center, University Hospital Hamburg-Eppendorf, Hamburg, Germany

**Keywords:** Marine chemistry, Prostate cancer

## Abstract

New bicyclic guanidine alkaloid, urupocidin C (Ur-C) along with the previously known urupocidin A (Ur-A) were isolated from the rare deep-sea marine sponge *Monanchora pulchra*, harvested in Northwestern Pacific waters. The unique structure of Ur-C was elucidated using 1D and 2D NMR spectroscopy as well as mass spectra. We discovered a promising selectivity of both alkaloids for human prostate cancer (PCa) cells, including highly drug-resistant lines, compared to non-malignant cells. In cancer cells, marine derived compounds were able to induce G1- and S-cell cycle arrest as well as caspase-mediated cell death. For the first time we have identified mitochondrial targeting as a central mechanism of anticancer action for these and similar molecules. Thus, treatment with the isolated alkaloids resulted in mitochondrial membrane permeabilization consequently leading to the release of cytotoxic mitochondrial proteins to cellular cytoplasm, ROS upregulation, consequent activation of caspase-9 and -3, followed by PARP cleavage, DNA fragmentation, and apoptosis. Moreover, synergistic effects were observed when Ur-A and Ur-C were combined with clinically approved PARP inhibitor olaparib. Finally, these alkaloids exhibited additive effects in combination with docetaxel and androgen receptor inhibitor enzalutamide, both applied in PCa therapy. In conclusion, urupocidin-like compounds are promising lead molecules for the development of new drugs for the treatment of advanced PCa.

## Introduction

Marine sponges are well-known producers of novel small molecules possessing unique chemical structures and bearing promising biological properties^1,2^. The need of self-defense against predators as well as competing species lead to the selection and accumulation of specific bioactive molecules (so-called secondary metabolites) used by the marine sponges and its symbiont organisms as a “chemical weapon” in this battle. The specific environmental conditions (low stable temperatures, high salinity and basic pH values of the ocean waters, lack of light etc.) stipulate a distinct biochemistry which leads to the production of secondary metabolites different from those biosynthesized by the terrestrial plants and animals^1^. Due to the limited access, marine organisms and their metabolites are less studied in comparison with terrestrial animals and plants. However, the number of studies reporting new marine derived compounds possessing promising bioactivities is growing annually^3^. To date approximately 10 drugs, which are based on small molecules or peptides isolated from marine organisms, are approved for clinical use – seven of those for anti-cancer therapy^3,4^. Additionally, many more are currently in different stages of clinical and preclinical development^4–6^. Marine sponges are considered as a primary source of many of these molecules as well as other materials like chitin and spongin, which are also used in biomedicine^7,8^. However, due to the high demand for these substances for further clinical development and use synthetic and biotechnological methods as well as aquaculture rearing have been established^9,10^.

Marine guanidine alkaloids are a group of biologically active compounds found in some marine sponges. These compounds were suggested to be chemotaxonomic markers of the marine sponges belonging to the genera *Ptilocaulis*, *Hemimycale*, *Crambe*, *Batzella*, *Clathria*, and *Monanchora*^11,12^. Specifically, guanidine alkaloids extracted from the marine sponges of the Monanchora genus (family Crambeidae) are characterized by unique chemical structures and an impressive spectrum of biological activities (reviewed in^11,13^). These molecules comprise pentacyclic, tricyclic, bicyclic or acyclic guanidine cores. In bicyclic alkaloids mono-, bi-, and trisubstituted skeleton systems can be found^14^. To date, only two members of the trisubstituted bicyclic guanidine alkaloids have been described, namely urupocidin A and B - both recently discovered by us^11,13–15^. In addition to its unusual structural features, urupocidin A (Ur-A) exhibits promising biological properties, including anti-cancer activities^15–17^. However, our knowledge about the mechanism of action of these newly identified alkaloids is still incomplete.

To date, cytotoxic activity of Ur-A has been reported against different human cancer cell lines^17^. Ur-A was found to inhibit an EGF-induced neoplastic transformation of epithelial cells^17^ and to induce a G_2_/M-phase cell cycle arrest as well as apoptosis of human cervical carcinoma HeLa cells. The latter was revealed by a significant increase of DNA fragmentation. However, only minor activation of caspase-3 and -7 was detected in the treated cells, suggesting a caspase-independent character of apoptosis induction^17^. In addition, Ur-A killed murine epithelial JB6 Cl41 cells without alteration of the transcriptional activity of p53, implying a p53-independent character of apoptosis^17^. Furthermore, Ur-A increased NO levels in murine macrophages treated at micromolar concentrations^15^. Finally, Ur-A and its semi-synthetic derivative inhibited TRPV receptors^16^. However, despite these findings, the current knowledge on the biological effects of marine natural compounds belonging to the urupocidin structural family is incomplete and cellular targets are still unknown.

In this study, in continuation of our search for bioactive natural products isolated from the northwestern Pacific marine sponge *Monanchora pulchra*^18^, we report on the isolation and structure elucidation of the new bicyclic guanidine alkaloid Ur-C along with previously known Ur-A. Mechanism of action and anticancer activity alone or in combination with established drugs were examined in human prostate cancer (PCa) cells. Prostate cancer was chosen because of the persisting high unmet medical need in advanced stages. In fact, aggressive phenotypes and impaired clinical outcome can be frequently found with increasing treatment duration due to development of resistance to standard therapies^19^. Both compounds showed cytotoxic activity in cell lines representing these advanced stages mainly mediated by mitochondrial targeting. Thus, Ur-A and Ur-C represent novel and promising candidates for development of effective drugs against aggressive and highly resistant PCa.

## Methods

### General procedures

Optical rotations of the purified compounds were measured using a Perkin-Elmer 343 polarimeter (Waltham, MA, USA). The ^1^H and ^13^C NMR spectra were recorded using a Bruker Avance III 700 spectrometer (Bruker, Bremen, Germany) at 700 (for ^1^H) and 175 (for ^13^C) MHz, respectively, with Me_4_Si as an internal standard. ESI mass spectra (including HRESIMS) were obtained using a Bruker maXis Impact II mass spectrometer (Bruker, Bremen, Germany) by direct infusion in MeOH. Low-pressure column liquid chromatography was performed using YMC*Gel ODS-A (YMC CO., LTD., Kyoto, Japan). HPLC was performed using an Agillent Instrument (Agilent Technologies, Santa Clara, USA) equipped with the differential refractometer and an YMC-ODS-A (250 × 10 mm) column (YMC CO., LTD., Kyoto, Japan).

### Animal material

The sponge *Monanchora cf. pulchra* (Lambe, 1894/1895) (class Demospongiae, order Poecilosclerida, family Crambeidae) was collected by dredging from a depth of about 139–141 m during scientific cruise onboard the research vessel “Academic Oparin” in May 2017 off Kunashir Island (45°52′3″ N; 149°37'0” E) and identified by Mr. Grebnev B.B. (G.B. Elyakov Pacific Institute of Bioorganic Chemistry, Vladivostok, Russia). A voucher specimen is kept under the registration number N 050-150 in the marine invertebrate collection of the G. B. Elyakov Pacific Institute of Bioorganic Chemistry (Vladivostok, Russia).

### Isolation of urupocidine A and C

After collection and prior to use the sample of the sponge *M. pulchra* was immediately frozen and stored at −20 °C. The compounds were extracted from the sample (sample dry weight 15.6 g) using EtOH (3 × 200 mL). The extract was concentrated *in vacuo* and the residue was separated by chromatography on a column with reversed-phase YMC Gel ODS-A sorbent, using EtOH-H_2_O (10: 90), and followed by EtOH-H_2_O (65: 35) + 0.1%TFA system as an eluent. The alkaloid mixture from the EtOH-H_2_O (10: 90) eluates was purified by repeated preparative HPLC with YMC ODS-A column using EtOH-H_2_O (62: 38) + 0.1%TFA system as an eluent to yield pure compounds **1** (15.3 mg, 0.098% of dry weight of the sponge) and **2** (91.9 mg, 0.59% of the dry weight of the sponge).

### Compound characterization data

Urupocidin C (**Ur-C**, compound **1**), a colorless glass; [*α*]^20^_D_ –22.8 (*с* 0.13, EtOH); for ^1^H and ^13^C NMR data, see Table 1. HRESIMS *m*/*z* 545.3802 [M + H]^+^, (calc. for C_29_H_49_N_6_O_4_ 545.3810), [M + 2H]^2+^; 273.1946 (calc. 273.1941).

**Table 1:** NMR data for urupocidin C (**Ur-C**, compound **1**; CD_3_OD).

No.	δ_H_, mult (*J* in Hz)	δ_C_	COSY	HMBC
1	0.91, t (7.4)	14.7 CH_3_	H2	C2, C3
2	1.37, m	24.5 CH_2_	H1, H3	C1, C3, C4,
3	2.03, m	30.8 CH_2_	H2, H4	C1, C2, C4, C5
4	5.39, m	131.9 CH	H3	C2, C3
5	5.39, m	130.6 CH	H6a, H6b	C6
6a	2.12, m	24.5 CH_2_	H5, H6b, H7	
6b	2.19, m		H6a	
7	1.55, m	39.8 CH_2_	H5, H6a	C6, C8, C9
8	3.77, m	70.0 CH	H7, H9	
9	1.91, m	39.0 CH_2_	H8, H9, H10	C8, C10
10	4.89, td (8.5, 3.0)	65.3 CH	H9, H11a, H11b	C11a
11a	2.35, m		H10, H11a, H12	C12, C13
11b	2.48, m	28.15 CH_2_	H10, H11b, H12	
12	3.59, m	33.7* CH_2_	H11a, H11b	C11, C14
13		168.5 C		
14		114.5 C		
15		180.9 C		
16	3.14, t (7.7)	39.0 CH_2_	H17	C14, C15, C17, C18
17	2.51, m	27.4 CH_2_	H16, H18	
18	5.44, m	129.4 CH	H17	
19	5.44, m	133.0 CH	H20	C20
20	2.03, m	30.8 CH_2_	H19	C19, C21
21	1.37, m	24.4 CH_2_	H20, H22	C20, C22
22	0.90, t (7.4)	14.7 CH_3_	H21	C20, C21
23		155.1 C		
24		165.3 C		
25	4.41, m	67.3 CH_2_	H26	C24, C26, C27
26	1.86, m	27.2 CH_2_	H25, H27	C27
27	1.86, m	24.7 CH_2_	H26, H28	
28	3.64, m	52.7 CH_2_	H27	C29
29		160.5 C		

Measured in CD_3_OD at 700 and 500 MHz. *Measured from HSQC and HMBC.

### Cell lines and culture conditions

Culture and handling of the cell lines was performed as described discribed previously^20^. Human prostate cancer cell lines 22Rv1 (androgen-independent, AR-V7(+)) and LNCaP (androgen-dependent, AR-V7(−)), as well as human fibroblast cell line MRC-9 were used. 22Rv1 and LNCaP cells were purchased from ATCC (Manassas, VA, USA). MRC-9 cells were kindly donated by Prof. Dr. Dr. med. Sonja Loges (University Medical Center Hamburg-Eppendorf, Hamburg, Germany). For 22Rv1 and LNCaP cells 10% FBS/RPMI medium was used (RPMI medium supplemented with Glutamax-I (Invitrogen, Paisley, UK) containing 1% penicillin/streptomycin (Invitrogen) and 10% fetal bovine serum (FBS) (Invitrogen)). For MRC-9 cells 10% FBS/DMEM medium was used (DMEM medium supplemented with Glutamax-I (Invitrogen) containing 1% penicillin/streptomycin (Invitrogen) and 10% FBS (Invitrogen)). The cell lines were recently authenticated by a commercial service (Multiplexion, Heidelberg, Germany).

### Analysis of apoptosis (annexin-V-FITC/PI double staining)

The experiment was performed as described before^21^. In brief, 0.2 × 10^6^ cells/well were seeded in 6-well plates, incubated overnight, and pre-treated with 100 µM z-VAD(OMe)-fmk or an equal volume of vehicle for 1 h in 2 mL of fresh media/well. Cells were then treated with the investigated drugs for 48 h, harvested with trypsin, immediately stained with propidium iodide and annexin-V-FITC for 15 min. Further analyses were performed using FACS Calibur (BD Bioscience, San Jose, CA, USA) followed by quantification using the BD Bioscience Cell Quest Pro v.5.2.1. software.

### MTT assay

The experiment was performed as described before^22^. In brief, 6000 cells/well were seeded in a 96-well plate, incubated overnight and treated with the tested drugs in 100 μl/well of fresh media for 48 h, unless otherwise stated. The cells were incubated with MTT reagent (3-(-4,5-dimethylthiazol-2-yl)-2,5-diphenyltetrazolium bromide) and the viability was measured using a spectrophotometer Infinite F200PRO reader (TECAN, Männedorf, Switzerland).

### Western blotting

The experiment was performed as described before^20^. In brief, cells (1 × 10^6^ cells/well) were seeded in ø 6 cm Petri dishes (TC Dish, Sarstedt, Numbrecht, Germany) in 5 mL/dish of media. The cells were incubated overnight and treated with the tested compounds for 48 h. Cells were harvested with the scraper, washed with ice cold PBS, and resuspended in Western blotting lysis buffer. The samples were frozen overnight at −20 °C, centrifuged at 10,000 g and the protein concentrations in the supernatants were determined using the Bradford assay. The total protein extracts (20–30 μg/ sample) were subjected to electrophoresis in gradient Mini-PROTEAN TGX Stain-Free gels (Bio-Rad, Hercules, CA, USA) at 200 V. The proteins then were transferred to a ø 0.2 μm pore PVDF membrane. The membrane was blocked with 5% BSA/TBS-T solution and then incubated with the primary antibody overnight according to the manufacturers‘ protocols. The membrane was washed with TBS-T, incubated with the secondary antibody, and again washed with TBS-T. The signal was detected using the ECL chemiluminescence system (Thermo Scientific, Rockford, IL, USA) according to the manufacturers‘ protocol. β-Actin was used as a loading control. For the list of used antibody see Supplementary information. The images were proceeded with the CorelDRAW X7 software (V. 17.1.0.572, Corel Corporation, Ottawa, Canada).

### Cell fractionation

The separation of cellular nuclear, mitochondrial and cytosolic fractions was performed using the Cell Fractionation Kit ab109719 (abcam, Cambridge, MA, USA) as reported before^21^. In brief, 4 × 10^6^ cells were seeded in T75 culture bottles containing 20 mL of media and incubated overnight. Cells were treated with investigated compounds for another 48 h and harvested using a cell scraper. Further procedures were performed according to the manufacturer’s protocol with slight modification. Note, cytosolic and mitochondrial fractions were concentrated using the Amicon Ultra-2 Centrifugal Filter device (Cat. No. UFC203024, Merck, Darmstadt, Germany).

### Analyses of cell cycle progression and DNA fragmentation

0.2 × 10^6^ cells/well were seeded in 6-well plates in 2 mL/well of media and incubated overnight. Cells were treated with the tested compounds for 48 h, harvested using trypsin and fixed in 70% EtOH/H_2_O (v/v) at −20 °C overnight. The cells were proceeded and stained with propidium iodide (PI)/RNase buffer and further analyses were performed using FACS Calibur (BD Bioscience, San Jose, CA, USA) followed by quantification using the BD Bioscience Cell Quest Pro v.5.2.1. software as described before^20^. Apoptotic cells (cells possessing fragmented DNA) were detected as sub-G1 population.

### Analysis of intracellular ROS level

Intracellular ROS levels were measured using the ROS-sensitive CM-H2DCFDA reagent (Cat. No. C6827, Molecular probes, Invitrogen, Eugene, OR, USA) as previously reported^21^. In brief, 0.1 × 10^6^ cells/well were seeded in 12-well plates with 1 mL media/well and incubated overnight. The culture media was exchanged with 0.5 mL/well of freshly made pre- warmed staining solution of 4 μM CM-H_2_DCFDA in PBS. The plates were incubated in the dark (30 min, 37 °C, 5% CO_2_), and the staining solution was exchanged with 1mL/well of pre-warmed PBS containing the investigated compounds or H_2_O_2_ (positive control). Cells were incubated for 2 h and then trypsinized, resuspended in 200 μL/sample of pre-warmed PBS and analysed by FACS Calibur device (BD Bioscience, San Jose, CA, USA) and BD Bioscience Cell Quest Pro v.5.2.1. software according to the manufacturer’s protocol.

### Analysis of mitochondrial membrane potential (ΔΨ_m_)

The alteration of the mitochondrial membrane potential (ΔΨm) was evaluated using ΔΨm-sensitive JC-1 dye (5,5′,6,6′-tetrachloro-1,1′,3,3′-tetraethyl-imidacarbocyanine iodide) as previously described^20^. In brief, 0.1 × 10^6^ cells/well were seeded in 12-well plates and incubated overnight in 1 mL media/well. The media was exchanged with fresh PBS (1 mL/well) containing the investigated compounds. The cells were incubated for 2 h (37 ° C, 5% CO_2_), trypsinized, pelleted and stained with 100 μL/well of 2 µM JC-1/PBS. The cells were further incubated for 1 h in the dark (37 °C, 5% CO_2_) and analysed using FACS Calibur device (BD Bioscience, San Jose, CA, USA) and BD Bioscience Cell Quest Pro v.5.2.1. software.

### Analysis of cytoplasmatic Ca^2+^ level change

Alterations of the cytoplasmatic calcium concentration were investigated by Fluo-4 NW Calcium Assay Kit (Cat. No. F36205, Molecular probes, Invitrogen, Eugene, OR, USA). In brief, 20 × 10^3^ cells/well were seeded in a black 96-well plate with transparent bottom in 100 µL media/well. The cells were incubated overnight and treated with investigated compounds in 100 µL fresh media/well for 2 h in the dark (37 °C, 5% CO_2_). The media was then removed, the plates were washed with PBS (100 µL/well), and 100 μL of the dye solution was added per well. The plates were incubated in the dark (37° C, 5% CO_2_) for 3 h, the dye solution was then exchanged by PBS (100 µL/well) and the measurement was performed using spectrophotometer Infinite F200PRO reader (TECAN, Männedorf, Switzerland) according to manufacturer’s protocol. The results were normalized against the cell viability, measured by MTS assay performed as described before^23^.

### Determination of cytotoxic effects in combination with clinically established drugs

Synergistic, antagonistic or additive effects of the isolated compounds in combination with cisplatin, carboplatin, docetaxel, olaparib and enzalutamide were evaluated by the Chou-Talalay method^24,25^. The data were generated using the MTT test as described above. The cells were treated with the individual drugs or their combinations in a non-constant molar ratio. A combinational index (CI) was calculated using the CompuSyn v.1.0 software (ComboSyn Inc., Paramus, NJ, USA). A CI > 1.2 indicates an antagonistic effect of the substances used in combination, a CI = 0.85~1.2 shows an additive effect and a CI < 0.85 indicates synergism.

### Statistical analysis

Statistical analyses of the generted data were proceeded with GraphPad Prism v. 5.01 software (GraphPad Prism software Inc., La Jolla, CA, USA). Data are presented as mean ± SEM. The unpaired Student’s t-test or one-way ANOVA followed by Dunnett’s post hoc tests were used for comparison of two or several groups, correspondently. The differences between the groups were considered to be statistically significant (*) if p <0.05. All experiments were performed in triplicates.

## Results

### Isolation of the compounds

**Figure 1:**
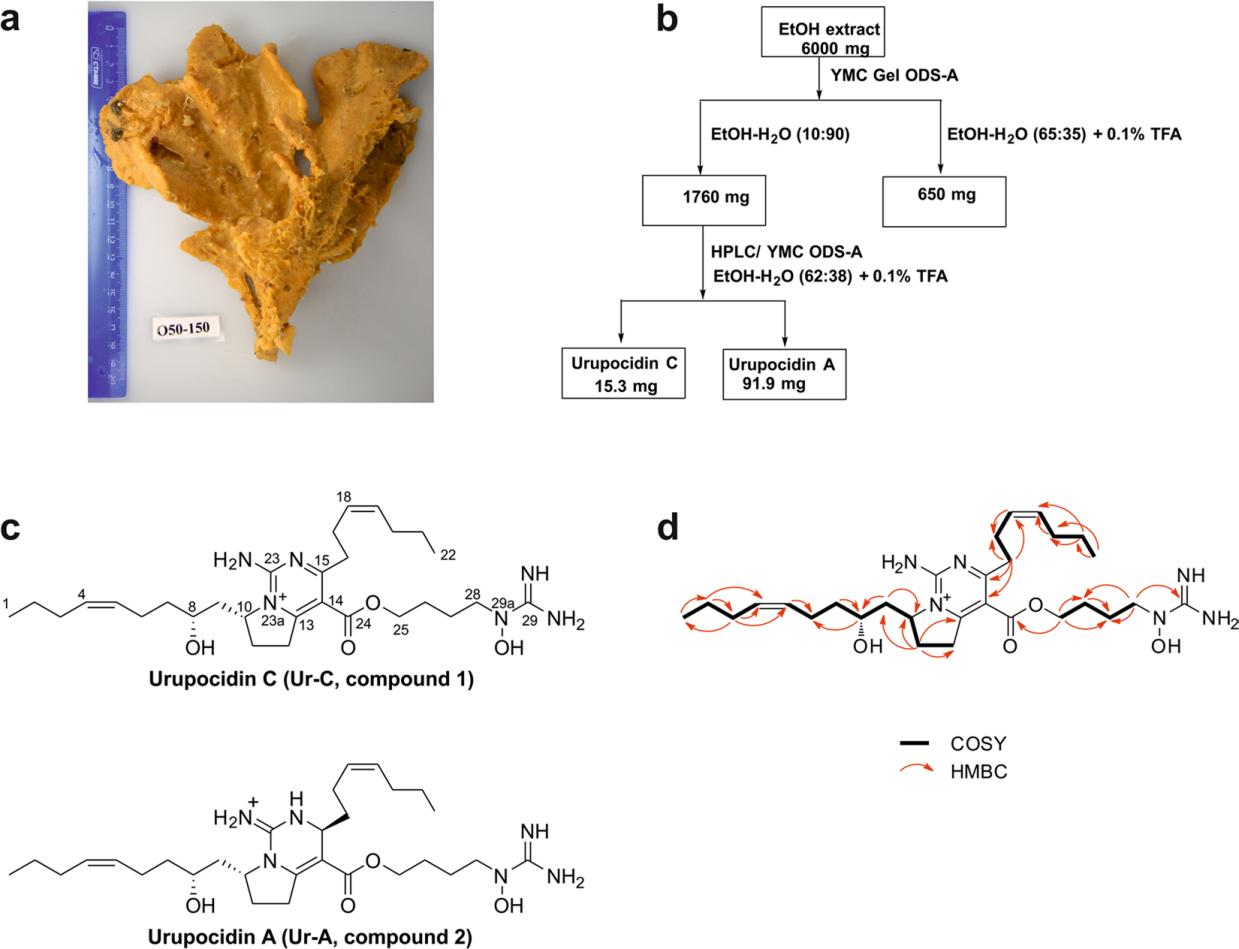
Marine sponge *Monanchora pulchra* (**a**). The schema of isolation (**b**) and the structures of urupocidin C (Ur-C, **1**) and A (Ur-A, **2**) (**c**). The key COSY (bold line) and HMBC (arrow line) correlations for Ur-C (**1**) (**d**).

The concentrated EtOH extract of the sponge *M. pulchra* (Fig. 1a) was separated using a reversed-phase column chromatography and the elution systems [EtOH: H_2_O (1:9)] → [EtOH: H_2_O (65: 35) + TFA (0.1%)] resulting in several subfractions (Fig. 1b). The subfraction eluted with [EtOH: H_2_O (1: 9)] was further purified using a reversed-phase HPLC and the elution system [EtOH: H_2_O (62: 38) + TFA (0.1%)] to obtain the two pure compounds **1** and **2** (Fig. 1c).

### Elucidation of the chemical structure

Compound **2** was identified as the previously known **Ur-A** based on its NMR and HRESIMS data and a comparison with the authentic sample of the previously isolated compound^15^ (Fig. 1c).

The molecular formula of the compound **1**, C_29_H_49_N_6_O_4_, was established from the [M + H]^+^ ion peak at *m*/*z* 545.3802 and [M + 2H]^2+^ ion peak at *m*/*z* 273.1946 in the (+)-HRESIMS. NMR data (Table 1) of compound **1** revealed the presence of signals, corresponding to resonances of two guanidine groups (δ_C_ 155.1 and δ_C_ 160.5), two methyl groups (δ_H_ 0.91/δ_C_ 14.7 and δ_H_ 0.90/δ_C_ 14.7), two disubstituted double bonds (δ_H_ 5.39/δ_C_ 131.9 and δ_H_ 5.39/δ_C_ 130.6; δ_H_ 5.44/δ_C_ 129.4 and δ_H_ 5.44/δ_C_ 133.0), one *N*-substituted CH groups (δ_H_ 4.89/δ_C_ 65.3), one *N*-substituted CH_2_ group (δ_H_ 3.64/δ_C_ 52.7), one oxymethine group (δ_H_ 3.77/δ_C_ 70.0), one carbonyl-linked oxymethylene group (δ_H_ 4.41/δ_C_ 67.3), and thirteen other methylene groups. Inspection of the ^1^H and ^13^C NMR spectra of **1** (Table 1) suggested a close relationship with Ur-A (**2**). Further analysis of the COSY, HSQC and HMBC data (Fig. 1d) of **1** indicated that the structures of the aliphatic chains in **1** were the same as in **2**. The ester carbonyl carbon C-24 at δ_C_ 165.3 was assigned by HMBC correlations from H-25, which was also correlated to C-26 and C-27. The chemical shift value of CH_2_-28 at δ_H_ 3.64/δ_c_ 52.7 indicated a hydroxylated N-29a nitrogen atom of guanidine group in **1**^15^. However, the other carbon signals observed in the spectra of **1**, were not in agreement with those observed for **2**. Specifically, no N-substituted methine signal was observed, and the HMBC spectra showed the presence of nonprotonated carbons at δ_C_ 114.5, 168.5, and 180.9. The chemical shift value of proton at C-10 at δ_H_ 4.89 supported the protonated form of the tertiary N-23a in **1**^26^. The downfield signal at δ_C_ 180.9 along with the nine degrees of unsaturation suggested by the molecular formula, showed that compound **1** was a dehydro-analogue of Ur-A. HMBC correlations from the methylene protons at δ_C_ 3.14 (H_2_-16) to carbons at δ_C_ 114.5 and 180.9, as well as to C-17 and C-18, and from H_2_-11 to C-12 and C-13 confirmed this notion. The bicyclic guanidine portion of **1** is thus similar to the bicyclic guanidine structures found in dihydrocrambescin A2 418^27^ and dehydrocrambine A^28^.

Compounds **1** and **2** may be biosynthesized via the same pathway as both alkaloids were isolated from the same marine sponge; therefore, it is very likely that their absolute configurations are also identical. The specific rotation of **1** was similar to the reported value of compound **2**^15^. Furthermore, as the absolute configuration of **2** has been established by chemical transformations and CD^15^, the absolute configuration of **1** was suggested to be 8*R*, 10*R*. Thus, **1** was identified as a new analogue of bicyclic guanidine alkaloids belonging to the urupocidin structural family^15^, and named urupocidin C (**Ur-C**). To the best of our knowledge, this is the third member of guanidine alkaloid class possessing trisubstituted bicyclic skeleton.

Thus, the isolated compound **1** was identified as a new natural product **Ur-C**, compound **2** – as a previously known **Ur-A** (Fig. 1c).

### Ur-A and Ur-C exhibit cytotoxicity in human prostate cancer cells

**Figure 2:**
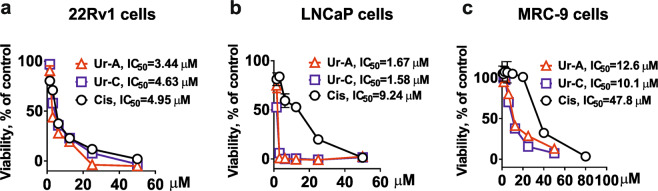
Effect of the compounds on viability of 22Rv1 (**a**), LNCaP (**b**) and MRC-9 (**c**) cells after 48 h of treatment. The viability was measured using MTT assay.

To investigate the cytotoxicity of Ur-A and Ur-C *in vitro*, we used the human castration resistant prostate cancer 22Rv1 cells (Fig. 2a), human hormone-sensitive prostate cancer LNCaP cells (Fig. 2b), as well as non-malignant human lung fibroblast MRC-9 cells (Fig. 2c). Cisplatin was used as a reference substance (positive control). The IC_50_s for both investigated compounds towards cancer cells were in the low micromolar range (Fig. 2a–c). For 22Rv1 cells the cytotoxic activity of Ur-C was comparable to cisplatin, whereas Ur-A was slightly more active (Fig. 2a–c). Notably, hormone-dependent LNCaP cells were ~6 times more sensitive to Ur-A and Ur-C in comparison to cisplatin (Fig. 2b). More important, the IC_50_s determined by MTT assay in cancer cells were significantly lower in comparison with non-malignant MRC-9 cells and the selectivity indexes (SI, MRC-9 versus 22Rv1 cells) for Ur-A and Ur-C were determined as 3.7 and 2.4, correspondently. Thus, we conclude both compounds to exhibit a selectivity towards human prostate cancer cells.

### Effect on cell cycle progression and apoptosis induction

**Figure 3:**
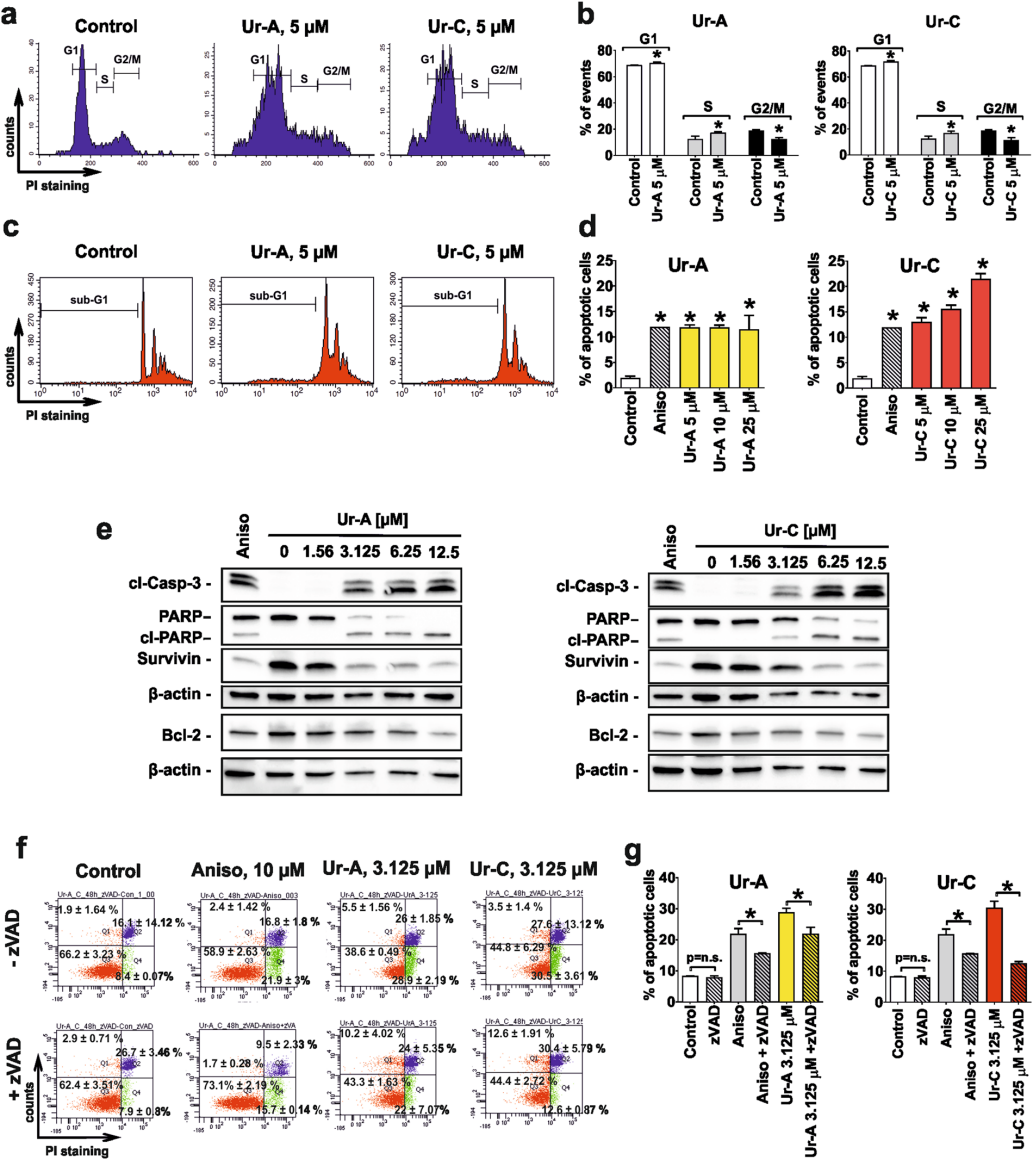
Flow cytometry analysis of cell cycle arrest (**a**,**b**) and DNA fragmentation (**c**,**d**). (**e**) Effect on pro- and anti-apoptotic proteins. The full-length blots are presented in Supplementary Fig. 1S. (**f**,**g**) Drug-induced externalization of phosphatidylserine and effect of pan-caspase inhibitor z-VAD(OMe)-fmk on it. The experiments were performed in 22Rv1 cells treated with Ur-A and Ur-C for 48 h. Flow cytometry data were quantified using the Cell Quest Pro software (**b**,**d**,**g**).

Drug-resistant 22Rv1 cells were chosen as a main model for the further investigation of mode of action of the isolated alkaloids. In order to investigate the mechanisms which stipulate the anticancer activity of the compounds, we further examined their effects on cell cycle progression and apoptosis induction in cancer cells. First, alterations of the cell cycle were accessed by flow cytometry (Fig. 3a). Cells treated with 5 µM of either Ur-A or Ur-C revealed significant increase of the cellular populations in G1- and S-phases, indicating a drug-induced cell cycle arrest (Fig. 3b).

In addition, a dose dependent DNA fragmentation of prostate cancer cells treated with Ur-A and Ur-C for 48 h was detected in flow cytometry indicating apoptosis induction (Fig. 3c,d). In line with this observation, Western blot analyses of the protein extracts of the treated 22Rv1 cells revealed dose-dependent PARP- and caspase-3 cleavage – two well-established apoptotic hallmarks (Fig. 3e). Furthermore, the expression of anti-apoptotic proteins, such as IAP protein survivin and Bcl-2 was suppressed under the treatment (Fig. 3e). Simultaneously, a phosphatidylserine externalization detected in the cells following 48 h treatment indicated the induction of a “classical” early apoptosis (Fig. 3f,g). Remarkably, induction of apoptosis was effectively inhibited by pre-treatment with pan-caspase inhibitor z-VAD(OMe)-fmk (Fig. 3f,g). In conclusion, these results indicate a caspase-dependent character of the Ur-A- and Ur-C-induced apoptosis in 22Rv1 cells.

### Apoptosis is exerted via intrinsic (mitochondrial) pathway

**Figure 4:**
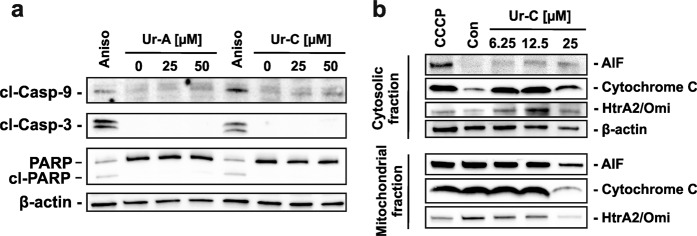
(**a**) Activation of caspase-9 prior to the cleavage of caspase-3 and PARP in 22Rv1 cells treated with Ur-A and Ur-C for 12 h. (**b**) Release of mitochondrial proteins to cytoplasm in 22Rv1 under treatment. The full-length blots are presented in Supplementary Figs. 2S and 3S.

Following the detection of caspase-3 activation as well as a caspase-dependent character of the induced apoptosis, we examined the effect of compounds on caspase-9. Remarkably, a cleavage of caspase-9 was detected following treatment with both, Ur-A and Ur-C. Of note, while an activation of caspase-9 was detected already after 12 h of treatment, no cleavage of caspase-3 was observed at this time (Fig. 4a). Therefore, we assume that treatment with Ur-A and -C first leads to an activation of initiator caspase-9, which later causes the cleavage of caspase-3 suggesting the intrinsic (mitochondrial) apoptotic pathway to be involved in the cytotoxic action of the compounds^29^.

To confirm this hypothesis we further investigated the effects on the mitochondria of the cancer cells. Mitochondrial stress is a primary event of the intrinsic apoptotic pathway, which is accompanied by mitochondrial membrane permeabilization and release of specific cytotoxic proteins^29^. Thus, we demonstrated a translocation of cytochrome C (an activator of procaspase-9) and the apoptosis inducing factor (AIF) from mitochondria to cytoplasm of treated 22Rv1 cells (Fig. 4b). This suggests the permeabilization of the mitochondrial membrane under treatment. In line with this, a down-regulation of the anti-apoptotic protein Bcl-2 was found (Fig. 3e). Bcl-2 down-regulation is known to cause release of apoptogenic proteins from mitochondria^30^.

### Mitochondria are a primary target of Ur-A and Ur-C in prostate cancer cells

**Figure 5:**
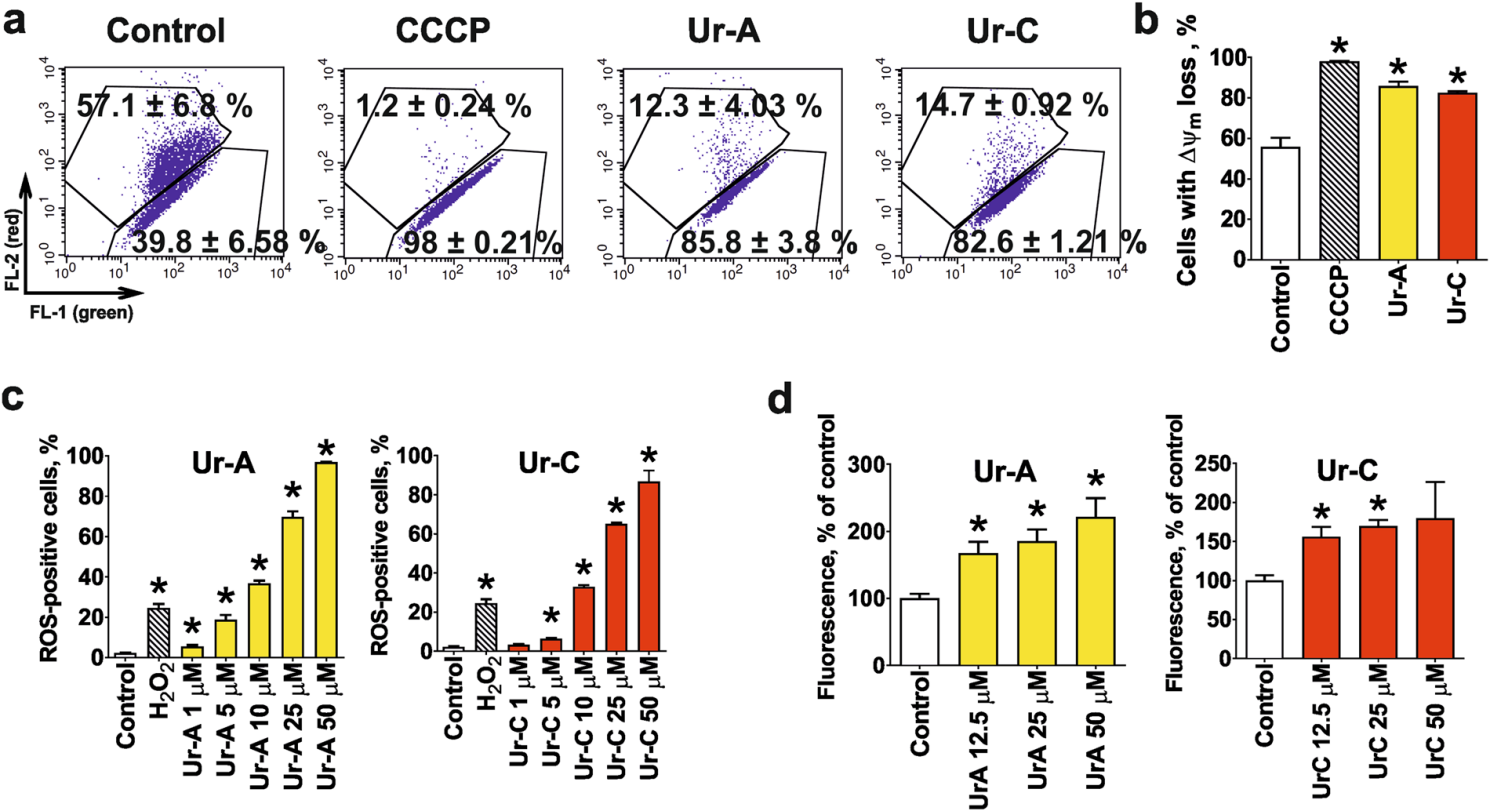
Induction of mitochondrial membrane potential (ΔΨm) loss (**a**,**b**), up-regulation of ROS (**c**), and induction of Ca^2+^-release from ER (**d**) in 22Rv1 cells following 2 h treatment with Ur-A and Ur-C. Flow cytometry data were quantified using the Cell Quest Pro software (**b**,**c**).

Based on the observations of the cleavage of caspase-9 prior to caspase-3, as well as the release of mitochondrial proteins to cytoplasm, mitochondria were assumed to be a primary target of Ur-A and Ur-C in cancer cells. For further confirmation, we investigated the initial effects, taking place in 22Rv1 cells shortly after treatment initiation. Interestingly, we could show that most of the cells exhibited the drop down of Δψ_m_ after 2 h treatment (Fig. 5a,b). Disturbance of mitochondrial integrity and permeabilization of mitochondrial membrane result in ROS induction and ROS production^31,32^. Indeed, an increase of the ROS level was detected shortly after drug exposure (Fig. 5c). Simultaneously, increase of the cytoplasmatic Ca^2+^ levels was observed (Fig. 5d). It is known that an elevated ROS level can lead to ER-stress and consequently to calcium release into cellular cytoplasm^33^. Therefore, the observed effect may result from ER targeting caused by ROS upregulation secondary to mitochondrial targeting.

### Anticancer *in vitro* effects of Ur-A and Ur-C in combination with established anticancer drugs

**Figure 6:**
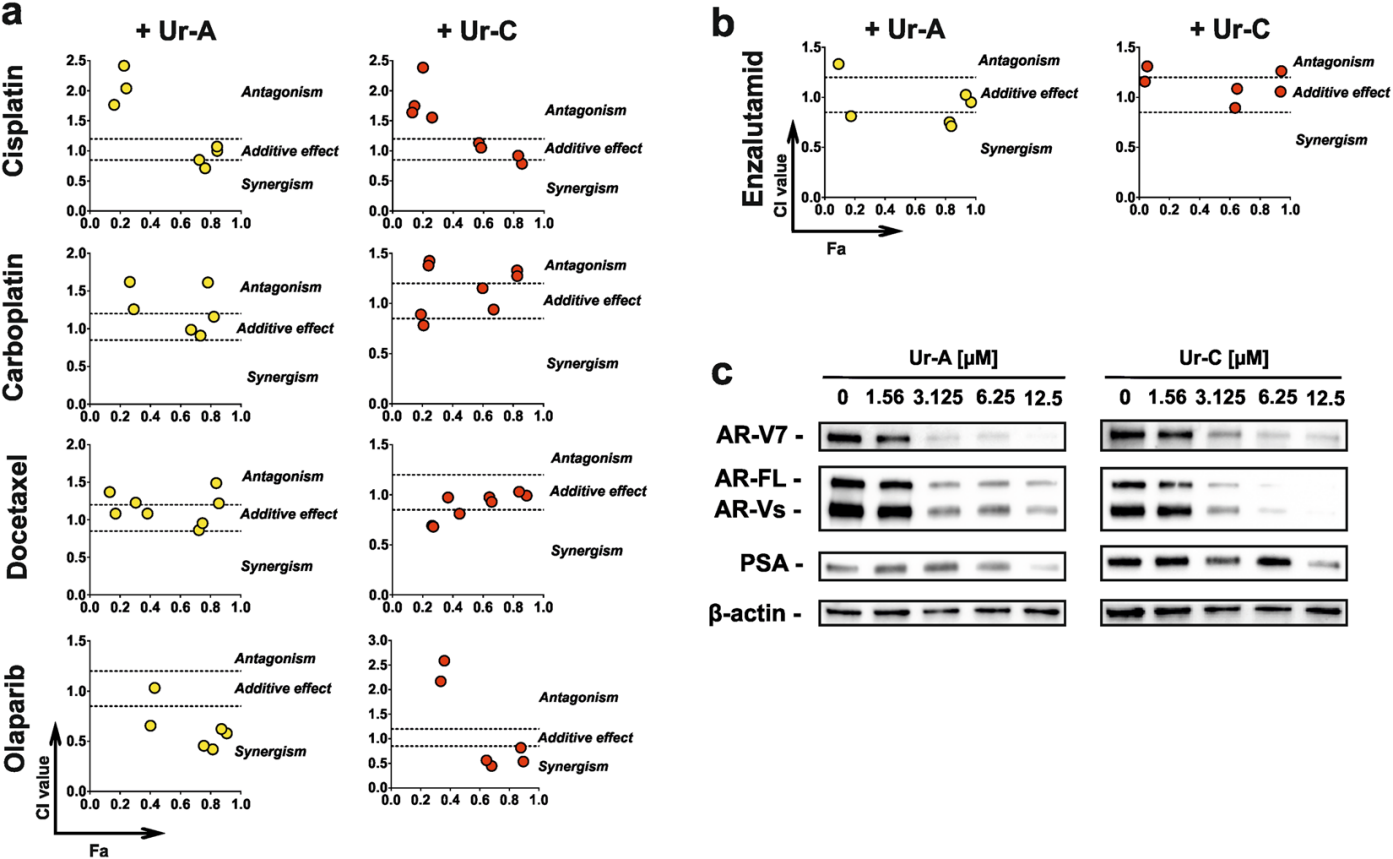
(**a**,**b**) Effects of Ur-A and Ur-C on cell viability in combination with established standard therapeutics. Data was generated using Chou-Talalay method and MTT assay. Effects were calculated using CompuSyn software. The molar ratio [Ur-A/C]: [Cisplatin] = 6.25: 10; [Ur-A/C]: [Carboplatin] = 6.25: 150; [Ur-A/C]: [Docetaxel] = 6.25: 0.02; [Ur-A/C]: [Olaparib] = 6.25: 100; [Ur-A/C]: [Enzalutamid] = 6.25: 100. (**c**), Treatment effects on AR-FL, AR-V7, and PSA expression. The experiments were performed in 22Rv1 cells treated for 48 h. The full-length blots are presented in Supplementary Fig. 4S.

The anticancer effects of Ur-A and Ur-C were examined in combination with standard anti-cancer therapies. Thus, we examined the effects of the isolated alkaloids together with DNA-binding (cross-linking) drugs cisplatin and carboplatin, microtubuline stabilizing agent docetaxel, PARP inhibitor olaparib (Fig. 6a), as well as androgen receptor targeting drug enzalutamide (Fig. 6b).

The combination of Ur-A and Ur-C with platinum based agents cisplatin and carboplatin showed additive effects in the range of high Fa (fraction affected) values, i.e. at cytotoxic doses of the combo drug (Fig. 6a). At the same time slight signs of antagonism were observed in the range of lower Fa values (Fig. 6a). This effect should be considered and carefully examined prior to further *in vivo* experiments or clinical trials. Combination of Ur-C with the taxol derivative docetaxel showed promising additive/synergistic effects, whereas the combination of Ur-A with docetaxel was less active (Fig. 6a). Most promising results were obtained for the combination with olaparib (Fig. 6a). For both Ur-A and Ur-C well pronounced synergistic effects were observed (CI <0.5, Fig. 6a).

Finally, additive effects of the isolated compounds in combination with anti-androgen drug enzalutamide were found (Fig. 6b). It is important to note that 22Rv1 cells used for the experiments express androgen receptor splice variant V7 (AR-V7)^34^, which can autoactivate the AR-pathway in the absence of androgens^35^ and ultimately lead to resistance to enzalutamide. Interestingly, we found a down-regulation/degradation of AR-V7 as well as AR full length (AR-FL) in cells treated with the investigated alkaloids (Fig. 6c), which could partially explain the observed recall of sensitivity to enzalutamide. In accordance with this, a down-regulation of PSA in treated cells was detected indicating a suppression of AR-signaling by Ur-A and Ur-C (Fig. 6c).

## Discussion

Mitochondria play an important role in energy metabolism, cell cycle regulation and survival of eukaryotic cells. The idea to develop molecules which are able to specifically target mitochondria raises from 1950 when the mitochondrial structure has been described and therefore the molecular basics of mitochondrial-targeting properties of small molecules have been first understood^36,37^. The concept of mitochondria targeting is based on the 3- to 5-fold difference between mitochondrial membrane potential (Δψ_m_) and plasma membrane potential^38^. Therefore, positively charged molecules, in particular of those containing so called delocalized lipophilic cations (DLC), are able to target and accumulate in mitochondria^36^.

Mitochondria are an attractive target for anticancer therapy. These organelles play a critical role in several apoptosis-related processes by mediating cytotoxic ROS production and release of cytotoxic proteins (e.g. cytochrome C, AIF, and others)^39^, which can be induced by a number of different drugs and stimuli. Additionally, they control vital ATP production and metabolic pathway signaling, which are critical for cell proliferation^40^. Thus, alteration of mitochondria function or homeostasis may easily initiate programmed cell death. Mitochondria of cancer cells exhibit special features which discriminates them from non-malignant cells. Thus, in some cases the mitochondria of cancer cells exhibit significantly higher transmembrane potential (Δψ_m_) in comparison to non-malignant cells^41,42^. This difference gives an opportunity to develop selective and effective drugs targeting cancer cell mitochondria and therefore the tumor cells^36^. Additionally, mitochondria targeting agents may cause less resistance in cancer cells due to the rather downstream position of this target in the apoptotic cascade^43^. Apoptotic cell death mediated by the mitochondria-targeting agents is often observed in fast proliferating cancer cells (due to the lower antioxidant capacity of mitochondria in these cells), while non-dividing or slowly proliferating cells are less affected or even intact^44^.

A well-known example for mitochondria-targeting is FDA-approved Bcl-2 inhibitor Venetoclax, which is used for treatment of recurrent leukemia and lymphoma^40^. Several other Bcl-2 family protein inhibitors are currently in different stages of clinical or preclinical development^40^. Additionally, standard chemotherapies such as paclitaxel, etoposide and vinorelbine may directly or indirectly affect mitochondria contributing to cancer cell death^39^.

In the current study, we isolated a new alkaloid Ur-C and previously known Ur-A from marine sponge *M. pulchra*. Along with urupocidins A and B^15^, Ur-C is the 3^rd^ known representative of trisubstituted bicyclic guanidine alkaloids. Ur-C exhibited a promising cytotoxic activity by targeting mitochondria of cancer cells. Thus, Ur-C and “mother” alkaloid Ur-A induced signs of mitochondria targeting such as Δψ_m_ loss, ROS production and ER stress shortly after treatment. This further lead to the release of cytotoxic mitochondrial proteins to cytoplasm, consequent caspase-9 activation, which further provoked caspase-3 and PARP cleavage, DNA degradation and ultimately resulted in caspase-dependent apoptosis. Both compounds also induced G1- and S-phase cell cycle arrest of 22Rv1 cells. Interestingly, we previously observed the G2/M-phase arrest in human cervical carcinoma HeLa cells treated with Ur-A^17^. Therefore, this effect seems to be cancer type specific (or even cell line specific). Accordingly, we did not detect any caspase-3 activation in the Ur-A-treated HeLa cells in our previous study^17^, whereas a cleavage of caspase-3 and moreover a caspase-dependent apoptosis was observed in prostate cancer cells within the current project.

Specific delivery of drugs to mitochondria (and therefore their targeting) may be provided by conjugation of mitochondria-targeting ligands (e.g. delocalized lypophilic cations, DLC) either directly to an active molecule or to a nanocarrier^39^. A unique feature of Ur-A and Ur-C is a guanidine moiety in the structure of both compounds. Guanidine belongs to the DLC family; it has a delocalized positive charge, which allows this molecule to enter the highly negative charged mitochondrial matrix^36^. Therefore, these bioactive alkaloids combine mitochondrial affinity and cytotoxic properties, which stipulates their mode of action and selectivity to cancer cells. Indeed, Ur-A and Ur-C revealed higher selectivity for PCa cells compared to non-malignant cells.

We observed a synergistic effect of the investigated alkaloids in combination with PARP (poly(ADP-ribose)polymerase) inhibitor olaparib. PARP detects ssDNA breaks and induces the repair cascade. A blocked repair of a single strand break (via PARP inhibition) causes more severe double strand breaks during DNA replication and consequently leads to apoptosis, which is also known as synthetic lethality^45,46^. Olaparib is effective in tumors harboring DNA repair deficiencies, especially those with double strand break repair defects, like BRCA1/2^47,48^. 22Rv1 cells are known to bear a BRCA2 mutation and therefore have an impaired dsDNA break repair mechanism making them sensitive to olaparib^49^. In our experiments, we have shown that treatment with Ur-A and Ur-C dramatically increases the intracellular ROS level. ROS are well-known inducers of ssDNA breaks^50^. Co-treatment with olaparib prevents a restoration of DNA, finally leading to dsDNA breaks. Due to the BRCA2 defect of 22Rv1 cells a so called synthetic cell death is ultimately induced. Of note, DNA repair defects are acquired during the course of treatment of advanced prostate cancer. Thus, while in localized disease only up to 3% of the patients harbor a BRCA2 defect, up to 13.3% carry this most common alteration in advanced stages of metastatic, castration resistant PCa^51^.

Finally, an additive effect was observed for the combination of Ur-A/C with the androgen receptor (AR) inhibitor enzalutamide. This anti-androgen drug binds to the AR thus blocking binding of this receptor to testosterone and dihydrotestosterone^49,52^. Thus, enzalutamide inhibits the AR-signaling cascade, which is essential for the survival and progression of prostate cancer cells^52,53^. However, in advanced disease stages prostate cancer cells may exhibit resistance to this drug. A major mechanism of resistance is alternative splicing leading to the expression of the androgen receptor splice variant V7 (AR-V7). AR-V7 lacks the C-terminal androgen binding domain, which leads to the permanent activation of AR as a transcription factor and results in the promotion of cancer cell growth^35,54^. We have demonstrated the ability of both Ur-A and Ur-C to increase the effect of enzalutamide in 22Rv1 cells, which are known to be resistant to enzalutamide-like AR-targeting agents due to the expression of AR-V7. Thus, the isolated alkaloids were able to re-sensitize the cells to AR-targeted therapy. This can be explained by the drug-induced decrease of AR-V7 expression, as well as general decrease of AR-signaling.

## Conclusions

Ur-C is a new cytotoxic bicyclic guanidine alkaloid, which was isolated by us from the deep-water marine sponge *Monanchora pulchra* along with the previously known Ur-A. The mechanism of action of both compounds includes mitochondria targeting, which further leads to the caspase-dependent apoptosis exerted via intrinsic pathway. Moreover, these alkaloids showed selectivity to human prostate cancer cells. Of high clinical impact is the observed synergism with olaparib as well as the ability to overcome AR-V7-mediated drug resistance to androgen receptor targeting drugs. Our current research significantly contributes to the understanding of the mechanism of action and therapeutic potential of urupocidins and similar compounds.

## Supplementary information

Supplementary Information.
