# Supplementary material for: Urupocidin C: a new marine guanidine alkaloid which selectively kills prostate cancer cells via mitochondria targeting
Source: Sci Rep. 2020 Jun 17;10:9764. doi: 10.1038/s41598-020-66428-5 (PMC7299949; doi:10.1038/s41598-020-66428-5)
Supplement: Supplementary file 1 — Supplementary Information. [file 41598_2020_66428_MOESM1_ESM.docx]

Supplementary information

Urupocidin C: a new marine guanidine alkaloid which selectively kills prostate cancer cells via mitochondria targeting

Sergey A. Dyshlovoy^1,2,3,4,#,^*, Ekaterina K. Kudryashova^2,#^, Moritz Kaune^1,#^, Tatyana N. Makarieva^2^, Larisa K. Shubina^2^, Tobias Busenbender^1^, Vladimir A. Denisenko^2^, Roman S. Popov^2^, Jessica Hauschild^1^, Sergey N. Fedorov^2^, Carsten Bokemeyer^1^, Markus Graefen^4^, Valentin A. Stonik^2^, Gunhild von Amsberg^1,4^

^1^ Department of Oncology, Hematology and Bone Marrow Transplantation with Section Pneumology, Hubertus Wald-Tumorzentrum, University Medical Center Hamburg-Eppendorf, Hamburg, Germany; dyshlovoy@gmail.com (S.A.D.), j.hauschild@uke.de (J.H.), moritz.kaune@stud.uke.uni-hamburg.de (M.K.), tobias.busenbender@gmx.de (T.B.), c.bokemeyer@uke.de (C.B.), g.von-amsberg@uke.de (G.v.A.)

^2^ G.B. Elyakov Paciﬁc Institute of Bioorganic Chemistry, Far-East Branch, Russian Academy of Sciences, Vladivostok, Russian Federation; catrinog.81@mail.ru (E.K.K.), makarieva@piboc.dvo.ru (T.N.M.), shubina@piboc.dvo.ru (L.K.S.), vladenis@piboc.dvo.ru (V.A.D.), prs_90@mail.ru (R.S.P.), fedorov@piboc.dvo.ru (S.N.F.), stonik@piboc.dvo.ru (V.A.S.).

^3^ School of Natural Sciences, Far Eastern Federal University, Vladivostok, Russian Federation

^4^ Martini-Klinik, Prostate Cancer Center, University Hospital Hamburg-Eppendorf, Hamburg, Germany; graefen@martini-klinik.de (M.G.)

^#^ These authors contributed equally

**Table S1.** The list of antibodies used.

| **Antibodies** | **Clonality** | **Source** | **Cat.-No.** | **Dilution** | **Manufacturer** |
| --- | --- | --- | --- | --- | --- |
| anti-AIF | mAb | rabbit | #5318 | 1:1000 | Cell Signaling |
| anti-AR | pAb | rabbit | sc-816 | 1:200 | Santa Cruz |
| anti-AR-V7 | mAb | rabbit | 198394 | 1:1000 | abcam |
| anti-Bcl-2 | pAb | rabbit | #2876 | 1:1000 | Cell Signaling |
| anti-cleaved Caspase-3 | mAb | rabbit | #9664 | 1:1000 | Cell Signaling |
| anti-cleaved Caspase-9 | mAb | rabbit | #20750 | 1:1000 | Cell Signaling |
| anti-cytochrome C | mAb | rabbit | #11940 | 1:1000 | Cell Signaling |
| anti-HtrA2/Omi | mAb | mouse | NB100-56558 | 1:250 | Novus biologicals |
| anti-mouse IgG-HRP |  | sheep | NXA931 | 1:10000 | GE Healthcare |
| anti-p21^Waf1/Cip1^ | mAb | rabbit | #2947 | 1:1000 | Cell Signaling |
| anti-PARP | pAb | rabbit | #9542 | 1:1000 | Cell Signaling |
| Anti-PSA/KLK3 | mAb | rabbit | #5365 | 1:1000 | Cell Signaling |
| anti-rabbit IgG-HRP |  | goat | #7074 | 1:5000 | Cell Signaling |
| anti-Survivin | pAb | rabbit | NB500-201 | 1:1000 | Novus |
| anti-α-Tubulin | mAb | mouse | T5168 | 1:5000 | Sigma-Aldrich |
| anti-β-Actin-HRP | pAb | goat | sc-1616 | 1:10000 | Santa Cruz |

## Original Western blotting files


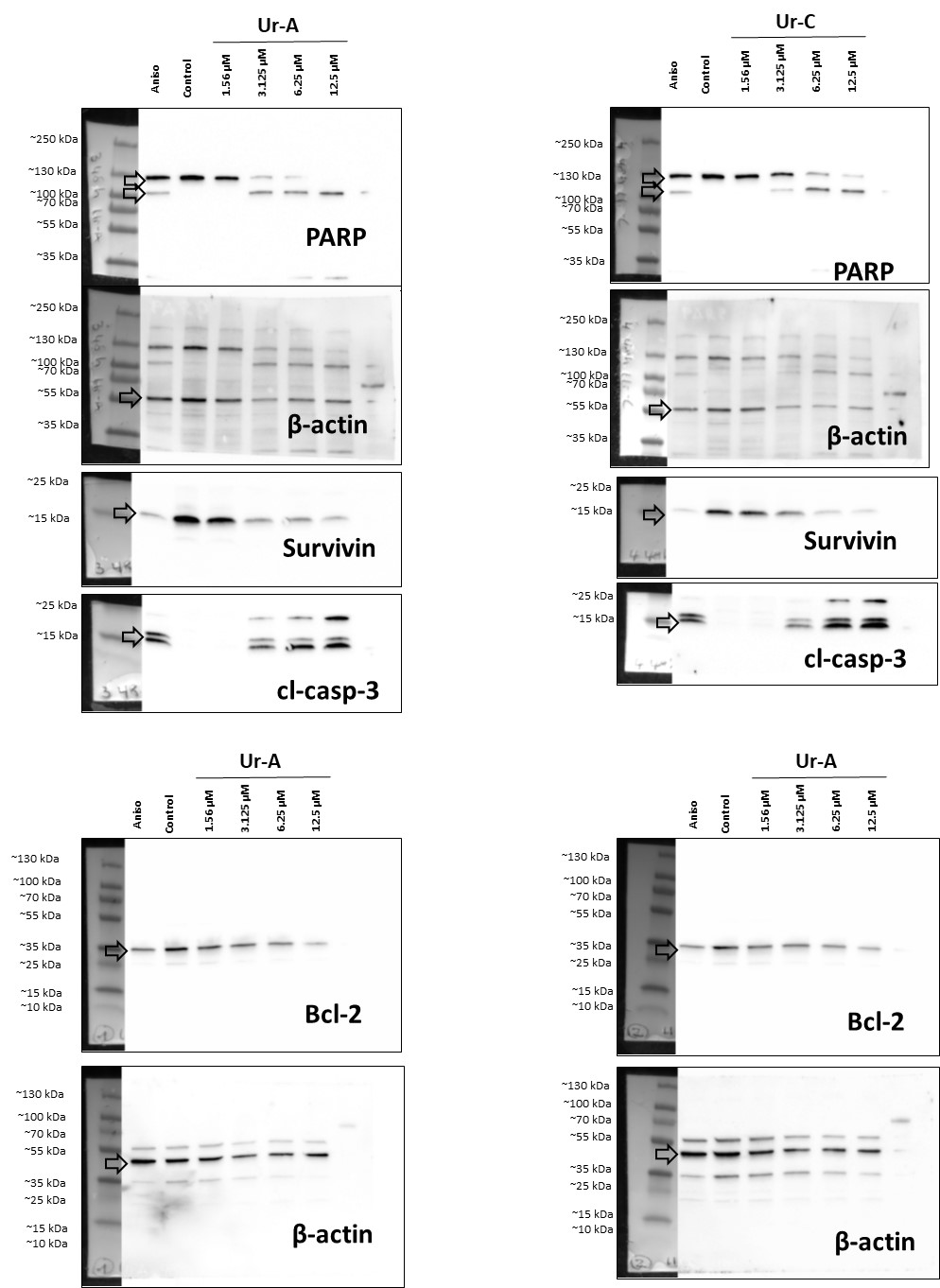


### **Figure 1S.** Original files for Figure 3e (Western blotting).


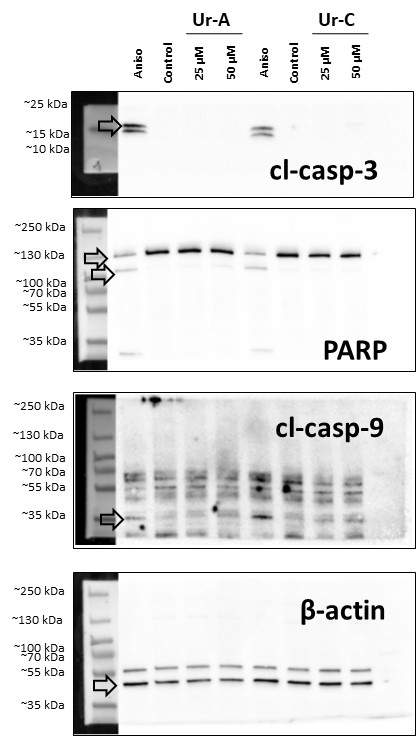


### **Figure 2S.** Original files for Figure 4a (Western blotting).


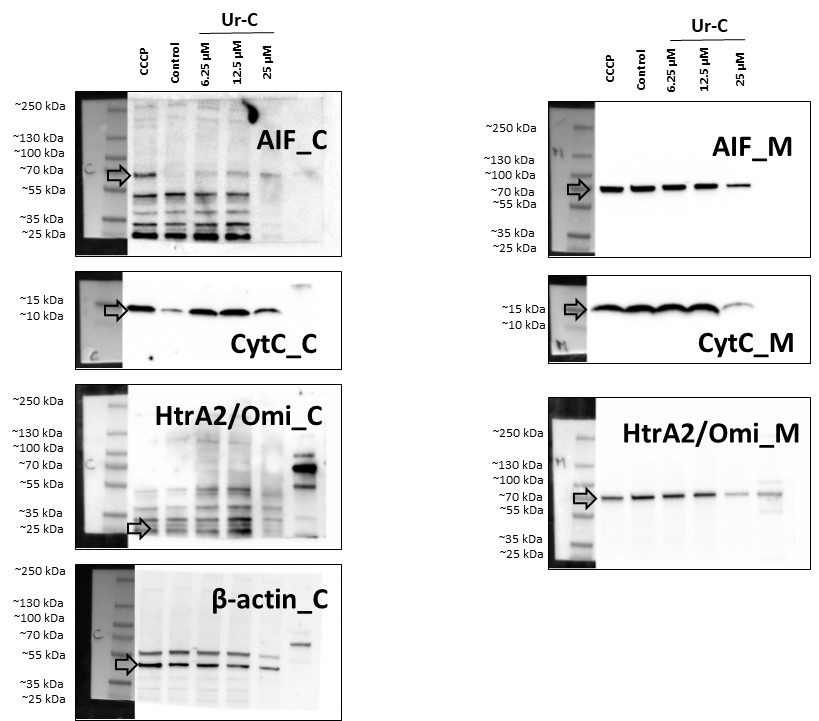


### **Figure 3S.** Original files for Figure 4b (Western blotting).


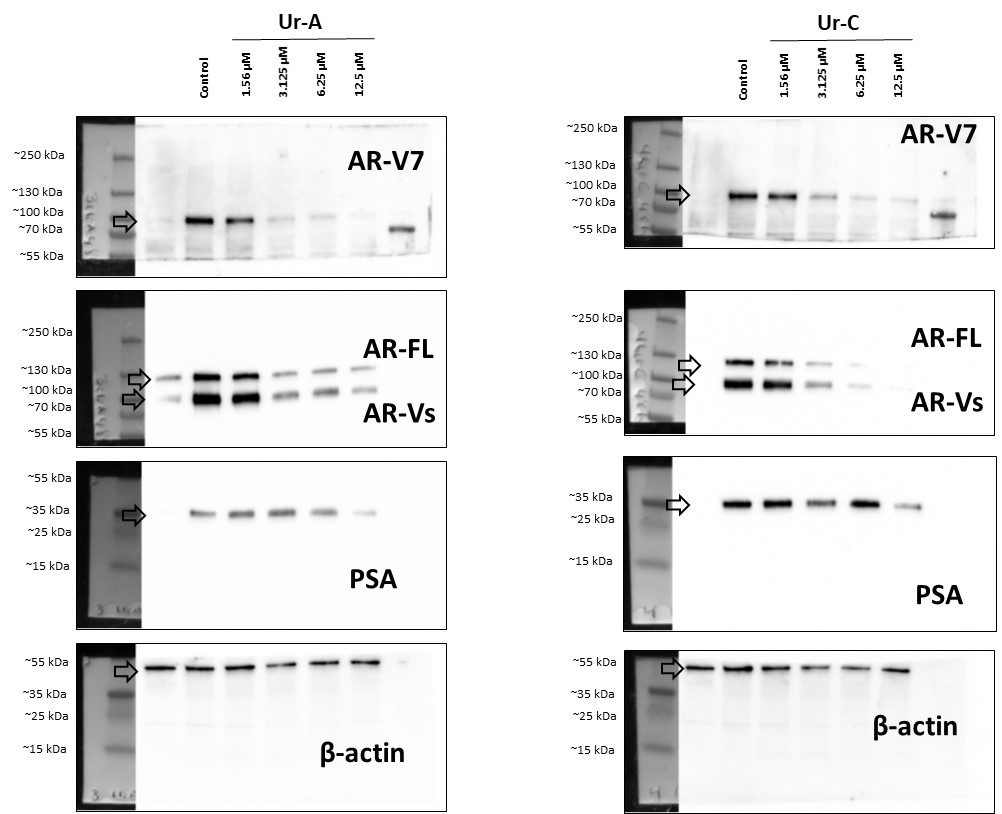


### **Figure 4S.** Original files for Figure 6c (Western blotting).
